# Effectiveness of a guided ACT-based self-help resilience training for depressive symptoms during pregnancy: Study protocol of a randomized controlled trial embedded in a prospective cohort

**DOI:** 10.1186/s12884-020-03395-9

**Published:** 2020-11-19

**Authors:** Anke B. Witteveen, Jens Henrichs, Annika L. Walker, Ernst T. Bohlmeijer, Huibert Burger, Yvonne Fontein-Kuipers, Francois G. Schellevis, Claire A.I. Stramrood, Miranda Olff, Corine J. Verhoeven, Ank de Jonge

**Affiliations:** 1 Department of Midwifery Science, AVAG/Amsterdam Public Health Research Institute, Amsterdam UMC, location VU University Medical Center, Van der Boechorststraat 7, 1081 BT Amsterdam, The Netherlands; 2 Department of Psychology, Health and Technology , University of Twente , Drienerlolaan 5, 7522 NB Enschede, Netherlands; 3 Department of General Practice and Elderly Care Medicine, University Medical Centre Groningen, Hanzeplein 1, Groningen, 9713GZ Netherlands; 4 Institute for Healthcare - School of Midwifery , Rotterdam University of Applied Sciences , Rochussenstraat 198, 3015 EK Rotterdam, Netherlands; 5 Department of General Practice , Amsterdam UMC Vrije Universiteit Amsterdam Amsterdam Public Health Research Institute , Van der Boechorststraat 7, 1081 BT Amsterdam, Netherlands; 6 NIVEL Netherlands Institute for Health Services Research , Otterstraat 118, 3513 CR Utrecht, Netherlands; 7 Department of Obstetrics and Gynaecology, OLVG, Oosterpark 9, 1091 AC Amsterdam, Netherlands; 8 Department of Psychiatry UMC, location AMC, Meibergdreef 9, 1105 AZ Amsterdam, Netherlands; 9 ARQ National Psychotrauma Centre , Nienoord 5, 1112 XE Diemen, Netherlands; 10 Department of Obstetrics and Gynecology Maxima Medical Centre , Veldhoven, Netherlands; 11 Division of Midwifery School of Health Sciences, University of Nottingham, Nottingham, United Kingdom

**Keywords:** Peripartum depression, Resilience, Psychological outcome, Infant development, Cortisol

## Abstract

**Background:**

During pregnancy, about 10 to 20% of women experience depressive symptoms. Subclinical depression increases the risk of peripartum depression, maternal neuro-endocrine dysregulations, and adverse birth and infant outcomes. Current treatments often comprise face-to-face psychological or pharmacological treatments that may be too intensive for women with subclinical depression leading to drop-out and moderate effectiveness. Therefore, easily accessible, resilience enhancing and less stigmatizing interventions are needed to prevent the development of clinical depression. This paper describes the protocol of a prospective cohort study with an embedded randomized controlled trial (RCT) that aims to improve mental resilience in a sample of pregnant women through a self-help program based on the principles of Acceptance and Commitment Therapy (ACT). Maternal and offspring correlates of the trajectories of peripartum depressive symptoms will also be studied.

**Methods:**

Pregnant women (≥ 18 years) receiving care in Dutch midwifery practices will participate in a prospective cohort study (n ~ 3500). Between 12 and 18 weeks of pregnancy, all women will be screened for depression with the Edinburgh Postnatal Depression Scale (EPDS). Women with an EPDS score ≥ 11 will be evaluated with a structured clinical interview. Participants with subclinical depression (*n* = 290) will be randomized to a 9-week guided self-help ACT-training or to care as usual (CAU). Primary outcomes (depressive symptoms and resilience) and secondary outcomes (e.g. anxiety and PTSD, bonding, infant development) will be collected via online questionnaires at four prospective assessments around 20 weeks and 30 weeks gestation and at 6 weeks and 4 months postpartum. Maternal hair cortisol concentrations will be assessed in a subsample of women with a range of depressive symptoms (*n* = 300). The intervention’s feasibility will be assessed through qualitative interviews in a subsample of participants (*n* = 20).

**Discussion:**

This is the first study to assess the effectiveness of an easy to administer intervention strategy to prevent adverse mental health effects through enhancing resilience in pregnant women with antepartum depressive symptomatology. This longitudinal study will provide insights into trajectories of peripartum depressive symptoms in relation to resilience, maternal cortisol, psychological outcomes, and infant developmental milestones.

**Trial registration:**

Netherlands Trial Register (NTR), NL7499. Registered 5 February 2019.

## Background

Antepartum depressive symptoms and subclinical depression with prevalence rates ranging from 10 to 20%, often remain unnoticed by maternity care providers during antenatal visits [1]. However, early detection is crucial because symptoms of depression or anxiety may result in antepartum depression with prevalence rates ranging from 5 to 13% or in postpartum depression (PPD) with prevalence rates of 10 to 15% [2, 3]. Because these depressive disorders are associated with reduced family functioning, impaired mother-to-infant bonding, adverse birth outcomes such as preterm birth, and cognitive and behavioral developmental delays in the infant, they form an essential treatment target [4–10]. A potential mechanism underlying these associations is stress-induced neuroendocrine dysregulation of the maternal Hypothalamic Pituitary Adrenal (HPA)-axis reflected by increased maternal cortisol levels that, in turn, affects the functioning of the neonatal HPA-axis [11].

An important protective factor against the development of depression is resilience: adapting to and recovering from stress [12]. Promoting resilience and positive mental health is a priority of the World Health Organization (WHO) [13]. However, little is known about resilience in the antepartum period and its preventive effect on maternal depressive symptoms and infant neurodevelopmental outcomes. In non-pregnant populations, positive associations between resilience and psychological well-being have been observed [12]. To date, only a few small studies have examined associations between levels of resilience (or related factors) and maternal depression and depressive symptoms or infant behavioral and developmental outcomes [14–16]. More specifically, by examining trajectories of antepartum and postpartum depressive symptoms in association with maternal antepartum resilience, psychological well-being, and developmental outcomes of the young child, a better understanding of individualized treatment options can be gained. Recently, researchers of a prospective cohort study (*n* = 2466) identified antepartum and postpartum risk factors (e.g., younger age, smoking, nausea, operative delivery, bonding difficulties, and low partner support) of several maternal depressive trajectories [17]. However, to date, additional examination of other psychological characteristics, including resilience and (chronic) neurobiological correlates associated with these trajectories, is lacking. New studies may provide evidence for relevant clinical management and treatment options for the near future.

Several international guidelines underscore the necessity to screen more adequately for antepartum depression or (subclinical) symptomatology. Guidelines also advise maternal healthcare providers to be aware that women may be unwilling to disclose their mental health issues and may be reluctant to engage in treatment due to fear of stigmatization [18, 19]. After detection of subclinical depressive symptoms, there is a clear need for effective interventions. Despite the advice for high-intensity psychological interventions such as cognitive-behavioral therapy (CBT) and interpersonal therapy [18], these interventions are less effective in women with subclinical antepartum or postpartum depression or show no effect at all in non-help-seeking women [20, 21]. An important problem of previous RCTs with high-intensity interventions to prevent perinatal depression are high attrition rates (e.g. on average 20%), likely because pregnant women are faced with practical demands (e.g., time constraints, lack of childcare), the stigma of experiencing psychological problems during pregnancy and physical issues [20]. From previous trials, we also know that to overcome high attrition rates and improve effectiveness, interventions should be aimed primarily at at-risk women (e.g., women with subclinical depression) and should fit the unique preferences and needs of pregnant women [22–24].

Psychological interventions that aim to improve mental resilience through self-help training might offer a less stigmatizing and more fruitful approach during pregnancy [25]. In general populations, a low level of antepartum resilience is associated with an increased risk of ante- and postpartum depression [26], while improving resilience has shown to prevent the development and recurrence of depression and other adverse outcomes such as PTSD [12, 27]. Interventions that aim to enhance resilience by improving stress-recovery in response to daily stressors, positivity, and flexibility such as Acceptance and Commitment Therapy (ACT) have already proven to be effective for non-pregnant individuals with subclinical depression [28]. Meta-analyses have shown that ACT, an innovative form of CBT, has a larger potential to induce psychological benefits and effectiveness in the long-term than classical CBT approaches [29, 30]. Although a four-session ACT-based group intervention in pregnancy has recently been developed [31], ACT’s effectiveness has not yet been evaluated among pregnant women with subclinical depression.

Exposure to (traumatic) stress before or during pregnancy is a significant risk factor for developing peripartum depression and anxiety and may result in less favorable outcomes for child development, family functioning, and physiological stress dysregulation [32–34]. During the early course of this (ongoing) research project, the COVID-19 pandemic unfolded and may have provoked additional prenatal stressors such as fear of getting infected, potential adverse health effects for the (unborn) baby, financial setbacks, and social distancing measures that may also affect maternity care. Research into natural disasters has shown that children in utero exposed to maternal prenatal stress display more adverse birth outcomes and infant developmental- and health outcomes [35, 36]. However, little is known about the potential buffering effect of maternal resilience on adverse effects of prenatal stress (due to e.g. COVID-19) on maternal mental health and infant development.

In this ongoing prospective cohort study, antepartum depression levels are measured in Dutch pregnant women (*n* = 3500). Subsequently, women with subclinical depression levels will be randomized to a guided self-help ACT-based resilience program or to care as usual (CAU). The short- and long-term effectiveness and feasibility of the intervention will be assessed using quantitative RCT data at multiple time-points and qualitative interviews. The prospective cohort data will be used to examine trajectories of different levels of peripartum depression and identify maternal psychosocial factors (i.e. stress and resilience) and demographic, neuroendocrine, and child-developmental factors that may characterize these trajectories.

## Method

### Study design

**Fig. 1:**
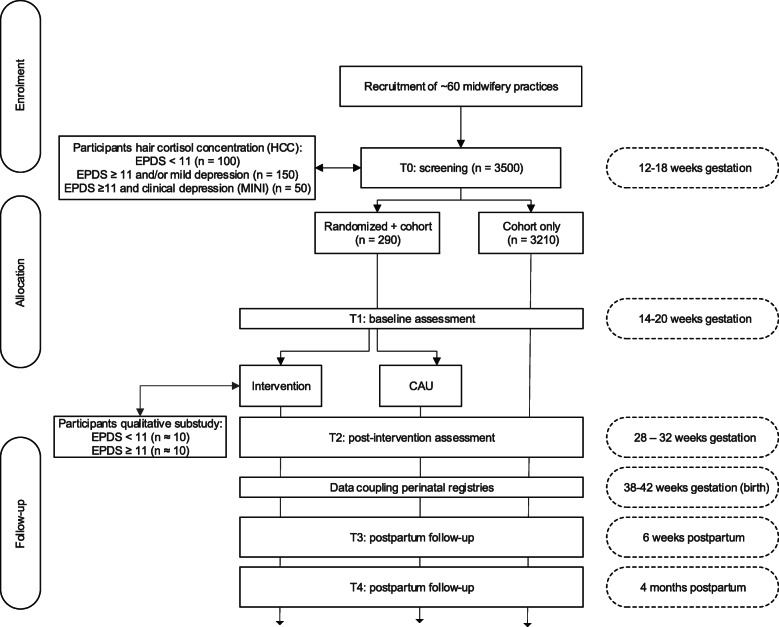
Flow-chart of the study

This study is designed as a prospective cohort study with an embedded randomized controlled trial (RCT) (Fig. 1). All women participating in the cohort study will be screened for depression at 12–18 weeks gestation. Women with subclinical depression will then be randomly allocated to either a guided self-help resilience program of 9 weeks (intervention) or care as usual (CAU) provided by maternity healthcare providers. All participants will be assessed at approximately 14–20 weeks (T1, RCT pre-intervention), at 28–32 weeks (T2, RCT one week post-intervention) gestation and postpartum at 6 weeks (T3) and 4 months (T4). Birth outcomes at T3 will be derived through data-linkage with the Dutch national perinatal registry [37]. Two additional sub-studies will be conducted to assess: (1) maternal cortisol levels (hair cortisol concentration (HCC)) in 300 participants randomly selected from the cohort study following T0, and (2) feasibility of the intervention via interviews with approximately 20 participants in the intervention group following T2. The Ethics Review Board of the VU University Medical Center has approved the study protocol (certificate number NL64740.029.18).

### Participants, eligibility, and screening

Women are eligible to participate when aged ≥ 18 years, receiving antepartum care at the moment of inclusion. Women will be excluded when they do not master the Dutch language sufficiently. After informed consent, all women will participate in a screening procedure for depressive symptoms (T0; 12–18 weeks gestation) and subsequent online assessments throughout pregnancy and postpartum (Fig. 1; T1 – T4).

For the embedded RCT, an additional inclusion criterion is an EPDS score of ≥ 11 [38] with or without a mild depressive disorder (i.e. a subclinical depression) as based on a structured clinical interview by telephone assessing the presence and severity of current depressive or dysthymic disorders and suicidality with the Mini International Neuropsychiatric Interview (MINI) supplemented with the Sheehan Disability Scale (SDS) [39–41]. The RCT’s exclusion criteria are psychopharmacological and/or psychological treatment over the past three months (reported by the participant), a current major depressive disorder, and/or moderate to high suicide risk (based on the MINI).

Additional inclusion criteria for the HCC sub-study are willing to provide a hair sample and sufficient hair growth at the posterior vertex of the head with a length of at least 1 cm. Women who used oral corticosteroids in the previous 3 months will be excluded from participation in the HCC sub-study.

For the qualitative sub-study, participants in the RCT will be purposively sampled from the intervention group to assess the self-help resilience training experiences. We will include a sample of ca. 20 women. The sample consists of women with improved EPDS scores (< 11) and women with continued depressive symptoms at T2 (EPDS score ≥ 11) as well as women who did or did not complete the full training and modules of the intervention.

### Recruitment

Since 87% of Dutch women receive midwife-led care at the onset of pregnancy, participants will mainly be recruited via midwifery practices (n ≈ 60) throughout the Netherlands. Midwives will inform eligible patients about the study (e.g., provide flyers) and ask women to be approached by a researcher through e-mail. Women are also recruited via short study leaflets and posters/flyers disseminated in waiting rooms, via advertising using social media, at pregnancy and baby shows/exhibitions, and directly via the study trial’s website. Women themselves or their midwives will submit the client’s contact-details on a secured access page of the study website. Subsequently, women will receive a complete subject information sheet and informed consent form via regular mail and e-mail with a charge-free return envelope. Women will be offered at least one week to consider their participation. If no response is received after two weeks, women will be sent an e-mail to remind them of the research and informed consent form.

### Study procedure

After providing and receiving informed consent, all participants will be screened for depressive symptoms through online completion of a digital version of the EPDS and, if the score is ≥ 11, followed by a 10–15 minutes telephone interview (MINI) to assess the severity of the depressive symptoms and to assess whether the participant meets DSM-5 criteria for current depressive or dysthymic disorders and suicidality. Master’s students in clinical or research psychology will conduct interviews. A healthcare psychologist with expertise in treating women in the antepartum and postpartum period will train the students.

In line with international guidelines for psychosocial care (NICE-Guideline, 2014) and from a medical-ethical perspective, all participants will receive feedback about their EPDS symptom level and, if applicable, about the psychiatric telephone-interview outcome. A copy of the two-step screening procedure results will also be shared with the participants’ midwife or obstetrician (for which participants gave informed consent). Midwives or obstetricians will be advised to discuss the outcome with their client and further monitor their client’s symptoms if screening results indicate a subclinical depression. If the MINI psychiatric interview identifies (suspected) major depressive or dysthymic disorder or suicidal ideations, they will be advised to refer women to specialized mental healthcare.

To randomly allocate pregnant women with subclinical depression to the intervention or control condition for the RCT, an independent statistician from the department of Epidemiology and Biostatistics (VUmc) will provide a computer-generated blocked randomization sequence in a 1:1 ratio, stored in individual sealed opaque envelopes. Block sizes will be undisclosed to ensure concealment. Following each RCT inclusion, a research assistant will open the next envelop and allocate the participant to the intervention or control condition. After randomization, participants in the control condition will be informed by mail that they will receive care as usual from their midwife or obstetrician. Participants randomized to the intervention condition will receive the ACT-based self-help book and its pregnancy adapted supplement.

After the screening procedure (T0), all study-participants will receive URL-links to the digital baseline- (T1) and follow-up questionnaires (T2-T4; Fig. 1). Each digital questionnaire assessment (T1-T4) will take approximately 30 minutes to complete. Researchers can analyze data collected through this web-based data-gathering tool without having access to information about the allocation.

For the HCC sub-study, a subsample of women will be asked to donate a hair sample at T1 (100 women with EPDS score < 11; 150 women with subclinical depression; 50 women with major depressive or dysthymic disorder based on the MINI). Hair samples will be collected at 14 to 20 weeks’ gestation. Due to COVID-19, partners (or other family members) will be asked to collect the participants’ hair samples at home. An instruction video will be shared for this purpose. Participants and their partners will be instructed to watch this video before hair sample collection to ensure a standardized hair sampling method. Hair strands of approximately 100 to 150 hairs will be cut as close as possible from the scalp from a posterior vertex position [42]. The most proximal 3 cm of hair will be used for laboratory analysis. Based on a mean hair growth rate of 1 cm per month, the hair samples reflect the cumulative cortisol and cortisone secretion of the previous 3 months. Participants of the HCC sub-study will be asked to provide additional written informed consent.

For the qualitative sub-study, a sub-sample of women from the intervention arm will be recruited via ‘purposively sampling’ approximately two weeks after the post-intervention assessment (T2). They will participate in a qualitative interview of 30–40 minutes (provided they have given additional consent). Interviews will be conducted using a topic-list with open-ended questions addressing the following substantive points during the interviews: (emotional) experience of the resilience-course, experiences of the e-mail coaching, working mechanisms, and point(s) of improvement of the intervention. Counselors will also be interviewed concerning the course of the intervention. A trained interviewer will conduct interviews. Results will provide important information for (process)evaluation, optimization, and future implementation of pregnant subclinically depressed women’s resilience course.

### Intervention

Participants in the intervention group will receive the ACT-based self-help book with 9 weekly modules [43]. They will also receive a pregnancy-specific supplement written by one of the developers of the ACT-based self-help book (author ETB) and two psychologists with expertise in perinatal psychology (authors ABW and JH). The supplement includes psychoeducation about depressive symptoms during pregnancy and explanations of ACT. The self-help book approach consists of acceptance, commitment, and mindfulness-based strategies and behavioral change strategies to increase psychological flexibility and resilience through six main ACT processes: acceptance, cognitive diffusion, being present, self as context, values, and committed action [44]. Each week, participants will follow one of the nine modules of the self-help book ‘Living to the Full’ [43]. The participants will reflect on their avoidance and control strategies and learn how to stop them and come into contact with their recent experiences. The book also focuses on raising awareness of significant personal values and subsequent decision making based on these values [45]. Each module takes about 2–3 hours a week (e.g., reading, homework, practicing). Daily mindfulness exercises of 10 to 15 minutes to reduce stress are encouraged and provided on audio. After each week, participants will receive motivational support through e-mails from trained counselors from the department of Clinical Psychology. Counselors will send an e-mail with questions about the progress during the previous week (e.g., ‘Were you able to perform the exercises in this module?‘). Participants are instructed to respond within two days, after which they receive a feedback e-mail from the counselor. The purpose of e-mail support (of approximately 10 minutes a week in total) is supporting participants in performing their homework and exercises and providing answers to questions about the self-help booklet [45]. The intervention condition and online counselors will be supervised by a clinical psychologist to monitor women’s possible worsening of symptoms. For the latter, symptom improvement or deterioration will be assessed every two weeks in both the intervention and control group with the Patient Global Impression of Change scale (PGI-C) [46].

### Care as usual

CAU comprises regular antepartum appointments with maternity healthcare professionals. Because of the RCT’s practice-oriented nature and ethical reasons, midwives can refer women of both the intervention and CAU groups for further psychological/psychiatric treatment if indicated and desired by the woman. All mental healthcare received by women from both RCT groups will be assessed at T2 (post-treatment). As a consequence of this study’s design, neither participants nor researchers can be blinded to group allocation. However, to avoid potential contamination, the study information letter initially provided to potential participants will not include details regarding the type of intervention (e.g., ACT [43]).

### Trial status

The cohort study with embedded RCT started enrolling on April 20, 2019. Currently, 757 participants have been enrolled in the cohort of whom 60 participate in the RCT.

## Assessments

### Primary outcomes

Depressive symptoms will be assessed with the 10-item Edinburgh Postnatal Depression Scale (EPDS) [47, 48]. The EPDS has been validated to screen for mild or major depression both antepartum and postpartum [49]. A Dutch validated EPDS version with good internal validity with a Cronbach’s alpha of 0.83 [48] and good concurrent and predictive validity is available [38]. The ten items’ response options range from 0 to 3, with higher scores indicating more depressive symptoms. For inclusion, a cut-off of ≥ 11 will be used since sensitivity and specificity for depression at this cut-off are estimated to be 0.83/0.90 in the antepartum period [18, 38, 50].

Resilience will be measured with the Dutch version of the Mental Health Continuum-Short Form (MHC-SF) (T2-T4; Table 1). Mental health or well-being acts as a resilience resource that buffers against psychopathology. The MHC-SF has 14 items that are scored on a 6-point scale ranging from 0 to 5 (never, rarely, sometimes, regularly, often, or (almost) always) and captures three dimensions: emotional (3 items), psychological (6 items) and social well-being (5 items) over the past 4 weeks. The Dutch version has shown good psychometric properties, is reliable (internal reliability of alpha 0.89 on MHC-SF total; and alpha’s of resp. 0.83, 0.83, and 0.74 on emotional, psychological, and social well-being subscales resp.) and was validated in a Dutch sample (n = 1621) [51]. With the MHC-SF, well-being can be distinguished into flourishing, neither flourishing nor languishing, and languishing (i.e., absence of well-being) [52].

**Table 1:**
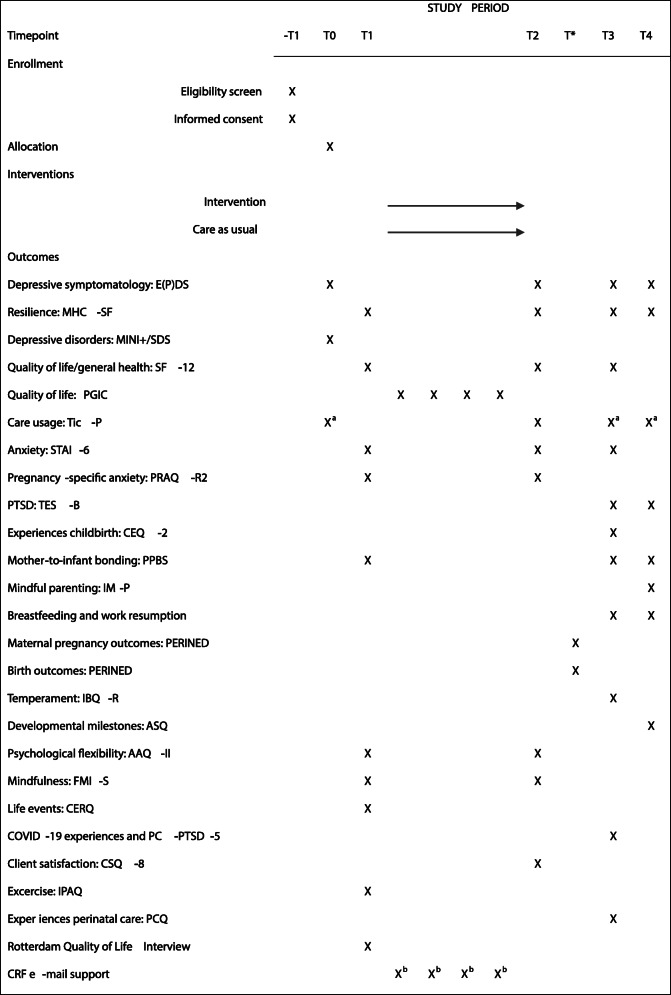
Measurements at each time-point of the study

T* birth outcomes PERINED data ^a^Part 2 of the Tic-P with questions regarding psychotropic drug use and psychosocial support. ^b^Case report form completed by e-mail counselors during intervention period

### Secondary outcomes

The following secondary outcomes will be obtained from participants. The exact time of administration can be found in Table 1.

- Quality of life will be assessed with the Dutch version of the Short-Form 12 item Health Survey (SF-12) [53] and during the intervention with the one-item version of the Patient Global Impression of Change scale (PGI-C) [46].
- Care use will be assessed with part two of the Trimbos/iMTA questionnaire of the Costs associated with Psychiatric Illness (TiC-P) [54].
- State anxiety will be assessed with the 6-item STAI-State (i.e. the STAI-S) [55]. The 6-item STAI-S was constructed and validated previously [56, 57].
- Pregnancy specific anxiety will be assessed with the revised 10-item version of the Pregnancy Specific Anxiety Questionnaire-Revised (PRAQ-R2) suitable for both primi- and multiparous women [58].
- PTSD symptoms will be assessed with the 24-item Traumatic Event Scale-B (TES-B) specified to childbirth as the traumatic event [59].
- Mother-to-infant bonding will be assessed with the 5-item Prenatal and Postnatal Bonding Scale (PPBS) [60].
- Experience of childbirth will be assessed with the 22-item Childbirth Experience Questionnaire (CEQ-2) [61].
- Questions concerning breastfeeding and work resumption (T3 and T4) are based on the DELIVER study and have been adapted by the research team [62].
- The Dutch 3 and 4 months version of the Ages and Stages Questionnaires-3 (ASQ-3) will be used to assess five infant developmental domains: Communication, Gross Motor, Fine Motor, Problem Solving, and Personal Social [63, 64].
- The revised version 37-item version of the Infant Behaviour Questionnaire (IBQ-R) will be used to assess the infant’s temperament, as reported by the mother. The IBQ-R items were slightly changed to make it available for assessment of 6-week old infants [65, 66].
- Obstetric variables such as birth weight and preterm birth will be extracted from the Perinatal Registry database of the Netherlands [37].

### Mediators/moderators

- Psychological flexibility will be assessed with the Dutch version of the 7-item Action and Acceptance Questionnaire-II (AAQ-II) [67].
- Mindfulness will be assessed with the Dutch version of the 14-item short form of the Freiburg Mindfulness Inventory (FMI-SF NL) [68].
- Coping with negative life events over the past year will be assessed with the Cognitive Emotion Regulation Questionnaire (CERQ) [69].
- COVID-19 related events experienced by (pregnant) women will be assessed with three closed questions about women’s health, the health of close relatives and financial setbacks, and three closed questions regarding the peripartum maternity care received.
- A potential (posttraumatic) stress reaction due to events like the COVID-19 crisis will be assessed with the Primary Care PTSD Screen for DSM-5 (PC-PTSD-5) validated in Dutch [70, 71].
- Satisfaction with obstetric care will be assessed with the 11-item Pregnancy and Childbirth Questionnaire (PCQ) validated in Dutch [72].
- Physical activity during pregnancy will be assessed with the short-form of the international physical activity questionnaire (IPAQ) [73].
- To assess satisfaction with the intervention, the Dutch version of the 8 item Client Satisfaction Questionnaire-8 will be used [74–76].
- To assess the (cumulative) cortisol concentration, hair samples will be weighted, cut with surgical scissors, and washed with 1.0 mL of LC-MS grade isopropanol for 2 min. The extraction of cortisol will be achieved by overnight incubation with methanol and analyzed with the LC-MS/MS grade including matrix interferences [77].

### Sociodemographic and medical variables

Sociodemographic and medical variables (T1) (e.g., smoking, alcohol use, age, parity, SES, ethnicity, gestational age, height and pre-pregnancy weight status, and work status) will be assessed with the pregnancy-adjusted Rotterdam Quality of Life interview [78] and using items derived from the DELIVER study [62].

## Statistical methods

### Power calculation

The medium effect (Cohen’s d) of ACT treatment on depressive symptoms lies between 0.4 and 0.6 [30, 79]. With a significant α level of 0.05 and 80% power we have to include 290 women to detect a small to moderate effect size (Cohen’s d) of > 0.33 between intervention and control group. Based on previous ACT-trials with similar (or even smaller) sample sizes of non-pregnant moderately depressed individuals, a Cohen’s d of at least 0.57 was found with largest Cohen’s d for women with increased depression levels at baseline [45, 80]. Assuming an attrition rate of 18% as in previous trials evaluating the long-term effectiveness of ACT [45, 80], the current sample-size allows us to detect small to moderate effect sizes (i.e., d = 0.365) with a power of 80% at the planned follow-up assessment [81]. We aim to include approximately 60 midwifery practices that will approach about 6,000 pregnant women, for participation. Based on an estimated response of 62% [62], we will be able to enroll approximately 3500 women in the cohort study of whom ca. 15% will have depressive symptoms [82]. Of these potential trial participants, 20% and 25% are expected to refuse participation or will be excluded, respectively, arriving at about 290 pregnant women with depressive symptoms to be included in the RCT.

### Statistical analyses

Analyses for the RCT will be done on both intention to treat (ITT) and per-protocol basis. First, to assess differences in depressive symptoms (EPDS) and resilience (i.e., well-being assessed with the MHC-SF) between intervention and control condition, independent t-tests will be used. The mean change of scores (T2-T1) will be standardized to interpret effect sizes in terms of Cohen’s d [83]. Next, ITT analyses will be performed using linear mixed modeling (LMM) to adequately deal with repeated measures data and clustering (i.e., at midwifery practice level). LMM’s with time, treatment, and time-by-treatment interactions will be performed to assess the intervention’s effectiveness in improving continuous primary outcomes of depressive symptomatology, resilience, and continuous secondary outcomes such as infant developmental milestones. Chi-square tests will be performed to assess group differences according to dichotomous (secondary) outcomes such as subclinical depression and flourishing incidence. Subsequently, generalized (binary) linear mixed models (GLMM) will be used to assess the intervention’s effect on the dichotomous secondary outcomes. To identify predictors of successful resilience training, (generalized) linear mixed models will be used. A multivariable GLMM to identify potential predictors of treatment success will be conducted in two steps: (1) univariable analyses to examine whether the potential predictor is significantly associated with the dichotomous outcome and (2) including significant predicting variables (*p* < .10) in the final multivariable model to identify independent predictors using the forward selection procedure. Finally, moderation and mediation analyses will be conducted to explore possible working mechanisms (psychological flexibility and mindfulness) of the intervention on maternal depressive symptomatology, resilience, and behavioral and infant developmental outcomes.

For the longitudinal data, latent class analyses will be used to explore different trajectories of depressive symptoms based on four repeated assessments over time. Correlates (e.g., cortisol levels) of perinatal depressive trajectories over time will be identified by using (generalized) linear mixed models. Associations between prenatal resilience and maternal mental health outcomes, birth, and infant developmental outcomes will be examined by using linear mixed models and GEE-analyses. All (main) analyses will be adjusted for potential confounders such as parity, educational level, and ethnicity. Multiple imputation methods will be used to impute missing values if missing at random can be assumed.

Audio-recorded qualitative interviews will be transcribed and anonymized. Transcripts of the interviews will be coded in an open, axial, and selective manner. Thematic analysis will be performed by two researchers [84]. After coding the first 3 or 4 interviews separately, both researchers will discuss the findings to modify the coding process. The software program MAXQDA will be used to facilitate the analysis process.

## Discussion

There is a lack of effective psychological interventions to reduce peripartum depressive symptoms and promote peripartum resilience. The present study is the first to examine the feasibility and effectiveness of a guided self-help ACT-based program for pregnant women with subclinical depression to decrease depressive symptoms and enhance mental resilience in the peripartum period. The RCT is embedded in a cohort study of pregnant women with follow-up assessments from early pregnancy up to 4 months postpartum. This cohort study explores the different trajectories of low to severe levels of depressive symptoms and examines the role of factors such as chronic stress, including maternal cortisol levels and resilience in relation to maternal mental health outcomes, birth- and infant developmental outcomes.

This study has several strengths: it will fill knowledge gaps concerning the effectiveness of interventions addressing the enhancement of resilience and the reduction of depressive symptoms among pregnant women with subclinical depression. If the ACT-based self-guided resilience training is shown to be effective, it will have important clinical implications as it is one of the first non-stigmatizing, easily accessible interventions for pregnant women. The ACT-based guided self-help intervention could fulfill an important role in the needed and strongly recommended prevention of peripartum clinical depression and its adverse consequences for both mother and child [85]. Using a self-help booklet with e-mail coaching may be particularly useful for women with mild to moderate depressive symptoms as it will probably result in better retention rates compared to face-to-face psychological or pharmacological interventions [20]. Furthermore, ACT has a unique transdiagnostic potential to affect a broader range of outcomes than depressive symptom reduction alone (e.g., anxiety and stress-related symptoms) and has shown to improve long-term outcomes even beyond the peripartum period [79].

Second, embedding this RCT in a prospective cohort study provides an excellent opportunity to examine trajectories of mental resilience factors and levels of peripartum depressive symptomatology in association with several maternal and child outcomes. To date, associations between resilience and psychological well-being have only been examined in non-pregnant populations, and associations between resilience factors and maternal or child outcomes have only been assessed in a few small-scale (longitudinal) studies [14–16].

Third, instead of cortisol from blood or saliva, antepartum hair cortisol levels will be assessed that are less invasive to obtain and will offer a more stable cumulative longitudinal measure of the maternal HPA-axis that can be linked to different levels of antepartum depressive symptoms and adverse maternal and child outcomes [86]. Furthermore, the association between antepartum resilience and chronic hair cortisol has only been studied once [87]. This study showed that pregnant women who were more resilient had lower hair-cortisol levels in the third trimester and less postpartum depressive symptoms, indicating the potential of resilience to prevent negative (mental) health outcomes in pregnant women and their offspring [87].

A limitation of this study is that multiple clinical self-assessments during the intervention period may reduce compliance and increase drop-out rates. However, we expect that continued participation will be encouraged through weekly positive interactions with the e-mail counselors. Compliance to fill out the baseline and follow-up assessments will be facilitated by using easily accessible online questionnaires. Another potential limitation is the maternal (and not paternal) report of infant developmental milestones, which might cause reporter bias, particularly in women with more depressive symptomatology.

In conclusion, the cohort data will deliver essential data to identify the role of resilience in relation to depression status during pregnancy and postpartum maternal and infant health. These longitudinal findings will also contribute to the development and strengthening of (existing) intervention strategies. With the embedded trial, the effectiveness of a pregnancy-adapted ACT-based self-help program in promoting resilience and reducing peripartum depressive symptoms will be evaluated. If successful, this will offer women and maternal healthcare providers a therapeutic option that is easy to incorporate into standard maternal care. Ultimately, it will also enhance more long-term resilience that may prevent postpartum mental health problems like depression, bonding difficulties, or postpartum traumatic stress.

## Data Availability

This is a study protocol; therefore, there are no data to share.

## Abbreviations

ACT: Acceptance and commitment therapy
CAU: Care as usual
HCC: Hair cortisol concentration
PPD: Postpartum depression
HPA-axis: Hypothalamic Pituitary Adrenal axis
CBT: Cognitive behavioral therapy (CBT)
